# Socioeconomic position and work, travel, and recreation-related physical activity in Japanese adults: a cross-sectional study

**DOI:** 10.1186/s12889-015-2226-z

**Published:** 2015-09-18

**Authors:** Munehiro Matsushita, Kazuhiro Harada, Takashi Arao

**Affiliations:** Faculty of Sport Sciences, Waseda University, Tokorozawa, Saitama Japan; Section for Motor Function Activation, National Center for Geriatrics and Gerontology, Obu, Aichi Japan

## Abstract

**Background:**

The aim of this study was to examine the association between socioeconomic position and the domains of physical activity connected with work, travel, and recreation in Japanese adults.

**Methods:**

A total of 3269 subjects, 1651 men (mean ± standard deviation; 44.2 ± 8.1 years) and 1618 women (44.1 ± 8.1 years), responded to an Internet-based cross-sectional survey. Data on socioeconomic (household income, educational level) and demographic variables (age, size of household, and household motor vehicles) were obtained. To examine the associations between socioeconomic position and physical activity, logistic regression analysis was used to calculate the odds ratio (OR) and confidence interval (CI) for “active” domains of physical activity.

**Results:**

Men with a household income of ≥7 million yen had significantly lower work-related physical activity than the lowest income group (OR 0.51; 95 % CI, 0.35–0.75), but significantly greater travel-related (OR 1.37; 1.02–1.85), recreational (OR 2.00; 1.46–2.73) and total physical activity (OR 1.56; 1.17–2.08). Women with a household income of ≥7 million yen had significantly greater recreational physical activity (OR 1.43; 1.01–2.04) than the lowest income group. Their total physical activity was borderline significant, with slightly more activity in the high-income group (OR 1.36; 1.00–1.84), but no significant differences for work- and travel-related physical activity. Men with higher educational level (4-year college or higher degree) had significantly lower work-related (OR 0.62; 0.46–0.82), and greater travel-related physical activity (OR 1.33; 1.04–1.71) than the lowest educated group, but there were no significant differences in recreational and total physical activity. Women with a 4-year college or higher degree had significantly greater travel-related physical activity than the lowest educated group (OR 1.49; 1.12–1.97), but there were no significant differences in any other physical activity. There was no relation between working full time and physical activity in men, but women working full-time have significantly lower and not higher travel related physical activity. (OR 0.75; 0.59–0.96).

**Conclusions:**

This study suggests that lower socioeconomic position is associated with more work-related physical activity, and less travel-related, recreational and total physical activity, and that this was more pronounced in men than in women.

## Background

Physical activity reduces mortality and the incidence of non-communicable diseases [1, 2]. However, a large proportion of the population in Japan and in many other countries remains physically inactive [3]. Promotion of physical activity is therefore a major public health concern globally. In Japan, the number of walking steps per day has decreased over time from around 1998–2000 [4], and a health promotion policy is needed to increase physical activity again. To increase participation in physical activity, it is necessary to understand the factors that influence this, and to design relevant policies and effective interventions based on that understanding.

Socioeconomic position (SEP), including for example, household income and educational level, is a determining factor of health status, and it is particularly important to promote a healthy lifestyle in low SEP populations [5]. In previous studies, lower SEP was associated with poorer health status and unhealthy behaviour among Japanese adults [6–8].

Studies that examined the association between SEP and total physical activity have, however, been inconsistent [9–13]. In a systematic review of European studies of the association between SEP and physical activity across the domains of work, travel and recreation, lower SEP was associated with greater occupational physical activity, but with less recreational physical activity [14]. It is also important to examine the association between SEP and domains of physical activity (DPA) such as work, travel, and recreation, to identify lifestyle factors that can be targeted to increase physical activity [15].

There is no comprehensive study examining the association between SEP and DPA among Japanese subjects. Japanese culture and lifestyles are different from those in Europe, so examining the relevant associations between socioeconomic position and physical activity in each domain of life in Japan is important for improving health promotion activity [16]. The association between SEP and health behaviours varies according to gender [7]; there are, however, few studies that have examined this [14]. The aim of this study was therefore to examine the association between SEP and DPA in Japanese men and women.

## Methods

### Participants and data collection

The data sample for this study consisted of 3269 respondents recruited to an Internet-based cross-sectional survey, which was conducted through a Japanese Internet research company, which had approximately 106,281 voluntarily-registered subjects across Japan in 2014. The company had access to detailed sociodemographic information and was able to target specific attributes. The set sample size and attributes for this study were: approximately 3000 adults, 30–59 years of age, stratified by age, gender, and household income distributions in line with the basic Japanese resident register for 2013, the 2012 Comprehensive Survey of Living Conditions in Japan [17, 18]. A total of 8284 potential respondents were randomly and blindly selected and subsequently invited via e-mail to participate in the Internet-based survey. There were 3269 respondents, a 39.5 % response rate. Of these, 137 were excluded because of missing data, leaving 3132 participants (final response rate: 37.8 %). The invitation e-mail contained a link directing the potential respondents to a protected area of the website where the questionnaire was located. They could then log on using their own login identity and password. As an incentive for participation, the respondents were offered reward points valued at 100 yen (1 U.S. dollar was approximately equivalent to 102 yen and €1 to 140 yen at the time). All the respondents voluntarily completed a demographic data information form and clicked on the “agree” button at the end of an online informed consent form approved by the Ethics Committee, Waseda University. The study received prior approval from the Ethics Committee, Waseda University, Japan.

### Measurements

#### Physical activity

The Global Physical Activity Questionnaire version 2 (GPAQ v2) is a questionnaire that was developed as a modification of the International Physical Activity Questionnaire for use in multi-ethnic settings by the World Health Organization [19]. GPAQ v2 was used to estimate the total weekly quantity (in minutes) of moderate-to-vigorous physical activity across three separate domains (work, travel, and recreation) lasting at least 10 min per session.

Previous study has shown that GPAQ has good test-retest reliability and validity [20]. Translation of the original content of GPAQ into Japanese, followed by back translation into English ensured the reliability of the questionnaire. No changes were made to the original content and wording of the questionnaire.

#### Demographic variables and SEP indicator

The demographic variables obtained from the research company included gender (men and women), age (categorized as 30–39, 40–49, and 50–59 years), size of household (0, 1, 2, 3, 4, and 5 or more people) and the number of household motor vehicles (none, 1 and 2 or more). Body Mass Index (BMI) was calculated from self-reported body height and body weight using the equation BMI = body weight in kg/body height in m^2^. The socioeconomic variables included household income (categorized as <3 million yen, 3–7 million yen, ≥7 million yen), educational level (junior high and high school graduation, 2-year college degree or equivalent, and 4-year college or higher degree), and employment status (non full time worker or full time worker).

### Data analysis

Data from 1579 men and 1553 women who provided complete information for the study variables were analysed. All the analyses were stratified by gender. Means, standard deviations and relative frequencies (%) were used to describe the subjects. To examine the associations between SEP and DPA, a logistic regression analysis was conducted to calculate the odds ratio (OR) and confidence interval (CI) for “active” physical activity. The dependent variable was physical activity. For the analysis, each DPA was categorised into “active” (physical activity performed at least 10 min duration during one or more days a week) or “inactive” (physical activity not performed at least 10 min duration during a week). And, total physical activity was categorised by WHO physical activity recommendation (active: ≥ 150 min/week, inactive: < 150 min/week) [21]. The independent variables included in the multiple binomial logistic regression analyses were household income, educational level and employment status with adjustment for age, size in household, BMI, and household motor vehicle ownership. Significance was set at a level of *p <*0.05. Analyses were conducted using SPSS 21.0 for Windows (SPSS Japan Inc., Tokyo, Japan, 2012).

## Results

### Basic characteristics of the respondents

Table 1 presents the demographic and socioeconomic data of the participants. A total of 3132 subjects, 1579 men (50.6 %) and 1553 women (49.4 %), participated in this study. The proportion in each class for age, size of household, and BMI were significantly different for men and women. The proportion who reported each class of household income (<3, 3–7 and ≥7 million yen) was 31.7, 40.7, and 27.5 % in men, and 30.9, 41.7 and 27.4 % in women. The proportion who reported each educational level class (junior high and high school graduation, 2-year college degree or equivalent, and 4-year college or higher degree) was 26.1, 16.3 and 57.6 % in men, and 26.7, 39.2 and 34.1 % in women. The proportion reporting each employment status (non full time worker or full time worker) was 23.7 and 76.3 % in men, 70.2 and 29.8 % in women. Men showed significantly higher work-related, recreational, and total physical activity than women.

**Table 1:** Characteristics of the respondents, stratified by gender

	General Japanese^a^	All		Men		Women		*p*-value
		*n* = 3132		*n* = 1579		*n* = 1553		
		n	%	n	%	n	%	
Age, years								
30–39	34.1	1072	34.2	540	34.2	532	34.3	0.919
40–49	35.0	1115	35.6	567	35.9	548	35.3	
50–59	30.9	945	30.2	472	29.9	473	30.5	
mean ± SD		44.2 ± 8.1		44.2 ± 8.1		44.1 ± 8.1		0.533
Living with family in household (no.)								
0 (live single)	32.4	555	17.7	365	23.1	190	12.2	<0.001
1	27.2	701	22.4	290	18.4	411	26.5	
2	18.2	841	26.9	414	26.2	427	27.5	
3	14.4	707	22.6	344	21.8	363	23.4	
4≥	7.8	328	10.5	166	10.5	162	10.4	
Body mass index, kg/m^2^								
<25	77.0	2478	79.1	1111	70.4	1367	88.0	<0.001
≥25	23.0	654	20.9	468	29.6	186	12.0	
mean ± SD		22.5 ± 3.8		23.6 ± 3.8		21.4 ± 3.5		<0.001
Household motor vehicles (no.)								
0	18.0	723	23.1	368	23.3	355	22.9	0.737
1	42.9	1385	44.2	705	44.6	680	43.8	
≥2	39.1	1024	32.7	506	32.0	518	33.4	
Household income								
<3 million yen	32.7	981	31.3	501	31.7	480	30.9	0.831
3–7 million yen	40.5	1291	41.2	643	40.7	648	41.7	
≥7million yen	26.8	860	27.5	435	27.5	425	27.4	
Educational level								
Junior high or high school graduation	65.3	827	26.4	412	26.1	415	26.7	<0.001
2 years college degree or equivalent	14.8	865	27.6	257	16.3	608	39.2	
4 years college or higher degree	19.9	1440	46	910	57.6	530	34.1	
Employment status								
Non full time worker	43.4	1465	46.8	375	23.7	1090	70.2	<0.001
Full time woker	56.6	1667	53.2	1204	76.3	463	29.8	
Physical activity								
Work								
Inactive	–	2614	83.5	1276	80.8	1338	86.2	<0.001
Active	–	518	16.5	303	19.2	215	13.8	
Travel								
Inactive	–	1724	55.0	877	55.5	847	54.5	0.573
Active	–	1408	45.0	702	44.5	706	45.5	
Recreation								
Inactive	–	2334	74.5	1127	71.4	1207	77.7	<0.001
Active	–	798	25.5	452	28.6	346	22.3	
Total								
Inactive	–	1737	55.5	818	51.8	919	59.2	<0.001
Active	–	1395	44.5	761	48.2	634	40.8	

*SD* Standard deviation

a: age, living with household, educatinal status, employment status: Basic Resident Register 2010; BMI: National Health and Nutrition Survey2012; Household motor vehicles: Market Trend Survey Passenger Vehicles 2013; Household income: Comprehensive Survey of Living Conditions 2012

### Household income and physical activity

Table 2 shows the associations between physical activity and household income in men and women, with adjustment for age, size of household, body mass index, household motor vehicle ownership, and educational level. Men with a household income of over 7 million yen had significantly lower work-related physical activity than the lowest income group (OR 0.51; 95 % CI 0.35–0.75), but significantly greater travel-related (OR 1.37; 95 % CI 1.02–1.85), recreational (OR 2.00; 95 % CI 1.46–2.73), and total physical activity (OR 1.56; 95 % CI 1.17–2.08). Women with a household income of over 7 million yen had significantly greater recreational physical activity than the lowest income group (OR 1.43; 95 % CI 1.01–2.04), but there was no significant difference regarding work- (OR 0.81; 95 % CI 0.52–1.25) or travel-related (OR 1.13; 95 % CI 0.83–1.53) physical activity. Total physical activity was borderline significant, with slightly greater activity in the high-income group (OR 1.36; 95 % CI 1.00–1.84).

**Table 2:** Socioeconomic inequalities in different domains of physical activity among Japanese men and women

	Work			Travel			Recreation			Total		
	Adjusted	95 % CI	*p*–value	Adjusted	95 % CI	*p*–value	Adjusted	95 % CI	*p*–value	Adjusted	95 % CI	*p*–value
	Odds Ratio			Odds Ratio			Odds Ratio			Odds Ratio		
Men												
Household income^a^												
<3 million yen	1.00			1.00			1.00			1.00		
3–7 million yen	0.82	(0.60 – 1.12)	0.216	1.14	(0.87 – 1.50)	0.334	0.93	(0.69 – 1.26)	0.655	1.04	(0.80 – 1.35)	0.782
≥7million yen	0.50	(0.33 – 0.73)	<0.001	1.44	(1.06 – 1.96)	0.021	1.89	(1.37 – 2.62)	<0.001	1.49	(1.11 – 2.01)	0.008
Educational level^b^												
Junior high or high school graduation	1.00			1.00			1.00			1.00		
2-year college degree or equivalent	0.83	(0.57 – 1.21)	0.323	1.26	(0.91 – 1.76)	0.165	1.09	(0.76 – 1.56)	0.641	1.21	(0.88 – 1.67)	0.237
4-year college or higher degree	0.60	(0.45 – 0.82)	0.001	1.34	(1.04 – 1.73)	0.026	1.17	(0.88 – 1.54)	0.281	0.99	(0.77 – 1.27)	0.937
Employment status^c^												
Non full time worker	1.00			1.00			1.00			1.00		
Full time woker	1.22	(0.88 – 1.68)	0.238	0.96	(0.74 – 1.25)	0.773	1.06	(0.80 – 1.42)	0.671	1.07	(0.83 – 1.38)	0.583
Women												
Household income^a^												
<3 million yen	1.00			1.00			1.00			1.00		
3–7 million yen	1.03	(0.71 – 1.48)	0.886	1.09	(0.84 – 1.42)	0.531	1.01	(0.73 – 1.39)	0.965	1.14	(0.88 – 1.49)	0.325
≥7million yen	0.79	(0.51 – 1.21)	0.277	1.23	(0.91 – 1.67)	0.180	1.58	(1.11 – 2.25)	0.011	1.40	(1.03 – 1.89)	0.032
Educational level^b^												
Junior high or high school graduation	1.00			1.00			1.00			1.00		
2-year college degree or equivalent	0.73	(0.51 – 1.05)	0.093	1.22	(0.94 – 1.60)	0.141	1.15	(0.83 – 1.58)	0.407	1.07	(0.82 – 1.40)	0.622
4-year college or higher degree	0.97	(0.66 – 1.43)	0.891	1.54	(1.16 – 2.05)	0.003	1.34	(0.95 – 1.88)	0.092	1.16	(0.87 – 1.54)	0.322
Employment status^c^												
Non full time worker	1.00			1.00			1.00			1.00		
Full time woker	0.84	(0.59 – 1.19)	0.321	0.75	(0.59 – 0.96)	0.021	0.96	(0.73 – 1.28)	0.795	0.97	(0.76 – 1.23)	0.800

*CI* confidence interval

^a^Adjusted for age, size of household, body mass index, household motor vehicle ownership, educational level, employment status

^b^Adjusted for age, size of household, body mass index, household motor vehicle ownership, household income, employment status

^c^Adjusted for age, size of household, body mass index, household motor vehicle ownership, household income, educational level

### Educational level and physical activity

Table 2 also shows the associations between physical activity and educational level in men and women, with adjustment for age, size of household, body mass index, household motor vehicle ownership, and household income. Men with a 4-year college or higher degree had significantly lower work-related physical activity than the lowest educated group (OR 0.62; 95 % CI 0.46–0.82), and higher travel-related activity (OR1.33; 95 % CI 1.04–1.71), but there were no significant differences in recreational (OR 1.29; 95 % CI 0.98–1.70) or total physical activity (OR 0.99; 95 % CI 0.78–1.26). Women with a 4-year college or higher degree had significantly greater travel-related activity than the lowest educated group (OR 1.49; 95 % CI 1.12–1.97), but there were no significant differences in work-related (OR 0.94; 95 % CI 0.64–1.37), recreational (OR 1.35; 95 % CI 0.97–1.88), or total physical activity (OR 1.19; 95 % CI 0.90–1.57).

### Employment status and physical activity

Table 2 also shows the associations between physical activity and employment status in men and women, with adjustment for age, size of household, body mass index, household motor vehicle ownership, and household income. Full-time working in men was not significantly related to physical activity, but women who worked full time had significantly lower travel-related physical activity (OR 0.75; 95 % CI 0.59–0.96).

## Discussion

This study is the first to examine the gender-linked association between SEP and physical activity in Japanese adults. In accordance with the previous systematic review of European studies [14], this study found various associations between SEP and different DPA in Japanese adults. There were also differences in these associations between the sexes. It is therefore important to take into account SEP, DPA, and gender when formulating proposals for the promotion of physical activity.

In work-related physical activity, the men with higher household income or educational status were less active than men with lower SEP. However, the relationship between SEP and work-related physical activity was not significant in women. Previous European studies had shown a consistent association between SEP and work-related physical activity in both sexes [14]. This difference in this study may be related to a difference in the assessment of work-related physical activity. Assessment of work-related physical activity in this study included housework, while previous studies had excluded this [22, 23]. Since 70.2 % of women in this study reported that they worked part-time (data not shown), most work-related physical activity for women in this study was likely to be housework. This could account for the lack of association between SEP and work-related physical activity in women.

In travel-related physical activity, the men with higher household income or educational status were more active than men with lower SEP. Also, the women with higher educational status were more active than women with lower educational status. However, full time employed women were less travel-related physically active than non-full time employed women The European review found no consistent association between SEP and travel-related physical activity in either sex [14]. In a Japanese study, Ishii et al. [24] reported that higher SEP was associated with more active commuting modes (e.g., walking, bicycle, public transport). In Japan, previous studies reported a significant association between the neighbourhood environment and travel-related physical activity [16, 24]. The significant direct association between SEP and travel-related physical activity found in this study might therefore be affected by environmental factors. However, the reasons for the differences between men and women in this study are unknown, and further study is therefore required.

In recreational-related physical activity, both men and women who showed high household income were more active than lower household income workers. There was also a direct association between SEP and recreational physical activity in both sexes in the European review [14], and earlier Japanese studies [7, 25]. Leslie et al. reported that high SEP residents had easier access to parks than lower SEP residents and used them more [26]. Psychological factors such as self-efficacy and self-control, and social factors, such as social interaction, mediate the relationship between SEP and recreational physical activity [27–29]. This study did not indicate why there was no significant association between educational level and recreational physical activity, though there was a trend towards an association in both sexes.

In total physical activity, both men and women who showed high household income were more active than lower household income workers. Previous Japanese studies have reported inconsistent associations between SEP and total physical activity [9–13]. Although high household income was associated with less work-related physical activity, this appears to be more than compensated for by greater travel and recreational physical activity.

This study had two major limitations. First, physical activity was not measured using an objective instrument. Objective measurements (using, for example, a pedometer) are generally recommended, but cannot provide data about physical activity in each domain. A self-report questionnaire was therefore used in this study to examine the association between SEP and the different domains of physical activity. It is therefore possible that the validity and reliability of GPAQ v2 is lower than an objective measure. However, GPAQ has been standardized and is used worldwide [19, 30].

Secondly, this study may have a sampling bias. Participants voluntarily registered with the Internet research company [31], and many Internet survey registrants expect an incentive from the research company. It is therefore possible that the study subjects were not representative of the local general population. This study may also have shown selection bias, since the response rate was lower in women, people who were younger and those with a lower income.

## Conclusions

Although this study has some limitations, our results suggest that both men and women with higher household income are more active in recreational-related and total physical activity than the both sexes with lower income. However, the relationships between SEP and work or travel-related physical activities are different in gender. To increase physical activity in Japanese adults with lower SEP, it will therefore be important to focus on increasing travel and recreational physical activity. In a follow-up study, the mechanism of the association between SEP and DPA will be examined.
